# Clinical characteristics and sensitivity to food and inhalants among children with eosinophilic esophagitis

**DOI:** 10.1186/1756-0500-7-47

**Published:** 2014-01-20

**Authors:** Erica Rodrigues Mariano de Almeida Rezende, Cristina Palmer Barros, Leandro Hideki Ynoue, Amanda Torido Santos, Rogerio Melo Costa Pinto, Gesmar Rodrigues Silva Segundo

**Affiliations:** 1 Department of Pediatrics, Universidade Federal de Uberlandia, Uberlandia, Minas Gerais, Brazil

**Keywords:** Esophagitis, Allergy, Sensitivity

## Abstract

**Background:**

To understand the clinical characteristics and the diagnostic procedures in pediatric patients with eosinophilic esophagitis and to evaluate the sensitivity of the patients to food and inhalant allergens. A cross-sectional study was performed in 35 children with eosinophilic esophagitis during the time period from January 2010 to January 2011. The clinical and epidemiological data were obtained using a questionnaire and medical chart analysis. The body mass index for age was used for the nutritional evaluation (via the Z score). The sensitivity to foods and inhalants was evaluated by performing a skin prick test and atopy patch test.

**Results:**

Patients (35 in total, median age 10 years) with a diagnosis of eosinophilic esophagitis were evaluated. The most prevalent symptoms in the patients were vomiting (71.4%) and abdominal pain (51.4%). Endoscopic alterations were observed in 97.2% of the patients. A good nutritional state was observed in 82.8% of the children. The tests demonstrated the presence of food sensitivities and/or aeroallergens in 27 (77.1%) patients, whereas 8 (22.9%) patients did not test positive in any of the tests performed. Among the patients with positive tests, 24 (68.5%) exhibited sensitivity to aeroallergens and 16 (45.7%) were sensitive to foods. The comparison between the sensitive and insensitive groups displayed statistically significant results with respect to sex, symptom prevalence, and 24-hour esophageal pH monitoring.

**Conclusions:**

The patients evaluated in this study displayed clinical characteristics of eosinophilic esophagitis similar to those reported in the literature. The sensitivity to foods determined by the tests was less than that observed in prior studies; however, a marked sensitivity to aeroallergens was observed. The different allergen sensitivity profiles observed in this study suggests that, similar to asthma, the eosinophiic esophagitis disease may exhibit several phenotypes.

## Background

Eosinophilic esophagitis (EoE) is defined as an immune-mediated chronic disease characterized by symptoms of esophageal dysfunction combined with the histological presence of an eosinophilic inflammatory process demonstrating at least 15 eosinophils (EOS) per high power field (HPF) in the absence of other eosinophilic syndromes [1,2]. Eosinophilic esophagitis was first described in 1978, and although the disease is considered rare, a substantial increase in the number of EoE cases has been reported in the last decade [3,4].

To diagnose EoE, it is necessary to exclude gastroesophageal reflux disease (GERD) as the cause of the esophageal eosinophilia. The GERD diagnosis is excluded by monitoring the pH acidity of the esophagus (24-hour pH monitoring), or following the failure of treatments with high doses of a proton-pump inhibitor (PPI) for at least 4 weeks, resulting in the persistence of eosinophils at greater than 15 EOS/HPF [1,2].

Little is known about the incidence of EoE. A prospective study performed in Denmark reported the prevalence as 0.16 cases of EoE per 10,000 inhabitants in that country [5]. In another study, an increase in the prevalence of the disease in Australian children was observed (from 0.05 to 0.89 cases per 10,000) [6]. A population survey performed in Sweden demonstrated the disease in approximately 1% of the population studied [7]. Another study reported that 8% to 10% of patients with GERD who were unresponsive to treatment with the PPI produced results compatible with a diagnosis of EoE [8].

EoE exhibits diverse clinical manifestations with the onset of symptoms at a variety of different ages. Infants and young children may display intolerance or may refuse food, or GERD symptoms may persist even after appropriate treatment [9]. Older children and adolescents may exhibit abdominal pain, vomiting, dysphagia, or impaction with solid foods [10].

Little is known about the pathogenesis of EoE, however, studies indicate similarities between the inflammatory process observed in the esophagus in EoE and that described for asthma and atopic dermatitis [11]. Several publications suggest that EoE is associated with food allergies, and clinical and histological improvements have been observed following the use of elemental diets [4,12].

Because of the lack of information regarding EoE, specially in Brazil, this study investigated the clinical characteristics of pediatric patients with EoE and evaluated the frequency of sensitivity to foods and inhalant allergens among the patients referred to our clinic.

## Methods

From January 2010 to January 2011, patients in the pediatric age range with a diagnosis of EoE and who were cared for at the Pediatric Gastroenterology and Food Allergy Clinic of the Clinical Hospital of Federal University of Uberlândia (HC-UFU) were invited to participate in this study. The exclusion criteria included the refusal to perform a phase of the study, patients who displayed a clinical improvement and histological normalization during the study period, or the presence of other chronic diseases (cystic fibrosis, cerebral palsy, chronic kidney disease, and severe heart disease). The guardians for the patients signed the informed consent forms.

The diagnosis of EoE was performed using the established consensual clinical and histological criteria, which consider the presence of more than 15 EOS/HPF in four biopsy samples [2].

A questionnaire was used for the clinical evaluation, and an assessment of the patient’s and the parent’s history of asthma, rhinitis, and atopic dermatitis was performed in addition to the analysis of data related to the date of diagnosis, endoscopy, biopsy, and 24-hour pH monitoring tests [13]. The patients underwent an anthropometric evaluation for weight and height for infants and evaluated according to body mass index for children after two years, using the Z score for age (WHO, World Health Organization) [14].

The endoscopies were performed by 2 pediatric endoscopists from the HC-UFU, and biopsies of the upper, middle, and lower thirds of the esophagus in addition to the stomach and duodenum were performed. For the diagnosis of EoE, in addition to the presence of 15 EOS/HPF in the histological evaluation of the esophagus, the biopsies performed in the stomach and duodenum did not indicate an above normal number of eosinophils at any location [2].

The patients underwent a 24-hour pH-monitoring exam. The adopted normalcy criterion was based on the reflux index and was considered acceptable when the esophageal pH was below 4 for less than 4.2% of the monitoring time [15]. All the patients were treated with the PPI (2 mg/kg/day) for 8 weeks. A new endoscopy was then performed, and a diagnosis of EoE was established when the histological evaluation showed a persistence of more than 15 EOS/HPF [2].

After filling out the questionnaire, the patients underwent a skin prick test (SPT) to evaluate their immediate sensitivity to foods and inhalants, and an atopy patch test was used to evaluate delayed sensitivities or sensitivities not measured using the IgE allergy test.

To perform the prick test, standardized allergenic extracts (Immuntech, Rio de Janeiro, Brazil) of the following aeroallergens were used: mites (*Dermatophagoides pteronyssinus*, *Dermatophagoides farinae*, and *Blomia tropicalis*), cockroaches (*Blattella germanica*), airborne fungi (*Alternaria sp., Aspergillus sp., Cladosporium sp.*, and *Penicillium sp.*), grasses, and dog and cat epithelia. The same procedure was used for the following foods: milk, egg white, soybean, wheat, corn, beef, pork, chicken, fish, oats, potato, beans, and rice. The positive control was performed using histamine chloride (10 mg/mL) diluted in physiological saline containing 50% glycerol, and the negative control was performed using the standard diluent used in the allergenic extracts. The test was read after 15 minutes by measuring the wheals (mm) using a graduated ruler. Papules with an average diameter measuring 3 mm larger than the negative control was considered prick test positive. The tests were performed by an appropriately trained experienced professional.

The food atopy patch test was performed using the following fresh foods: powdered skim cow’s milk, egg white, infant soy formula, wheat flour, corn, beef, pork, chicken, and saltwater fish. The meats were cooked in distilled water for 10 minutes before administration and were prepared at a dilution of 2 grams of food per 2 ml of isotonic saline solution [16]. An appropriately trained nutrition professional prepared and handled the food in an experimental kitchen at the HC-UFU, and a high precision scale was used. The extracts were placed on filter paper and were attached to aluminum chambers (Finn Chamber, Phoenix, United States) measuring 8 mm in diameter, which were adhered to the upper region of the patient’s back. The extracts were removed after 48 hours, and a reading was performed 72 hours after the material was put in place. The evaluation was performed by the same researcher and at the same location where the containers were placed following the technique described by Spergel et al. [16].

The Ethics Committee of Federal University of Uberlândia approved this study. The results were analyzed using descriptive statistics to characterize the group of evaluated patients, and to compare the sensitive and insensitive groups, the Mann–Whitney test was used with *p* < 0.05. The data were analyzed using the SPSS for Windows, version 17.0 software (SPSS, Chicago, IL).

## Results

Of the 45 patients with a diagnosis of EoE who were treated during this period, 10 were excluded; 4 were excluded because of inactivity of the disease, 5 because they suffered from cerebral palsy, and 1 for refusing to undergo the prick test.

Of the 35 patients who were analyzed, 20 (57.1%) were male. The median age was 10 years with a variance of 1.6 to 15.3 years. At diagnosis, the mean age was 7.4 years with a standard deviation of 3.8 years, and for the age at the onset of symptoms, as reported by the parents; the median was 2.3 years with a variance of 1 month to 13 years. A history of atopy in the patient and in the parent was observed in 18 (51.4%) of the evaluated patients. Good nutritional conditions were observed in 29 (82.8%) of the individuals. Underweight was observed in 2 (5.7%) of the patients, and overweight was observed in 4 (11.4%) of the patients. These data are shown in Table 1.

**Table 1:** Clinical characteristics of the patients with EoE treated at the HC-UFU

**Data**	**Values**
Median age in years (variance)	10 (1.6 a 15.3)
Male Sex (%)	20 (57.1)
Parental history of atopy (%)	18 (51.4)
Personal history of atopy (%)	
Asthma	21 (60)
Rhinitis	26 (74.2)
Atopic dermatits	15 (42.8)
Mean age at diagnosis (SD)	7.4 (3.8)
Nutritional status (%)	
Normal weight	29 (82.8)
Underweight	2 (5.7)
Overweight	4 (11.4)

*SD* standard deviation.

**Figure 1:**
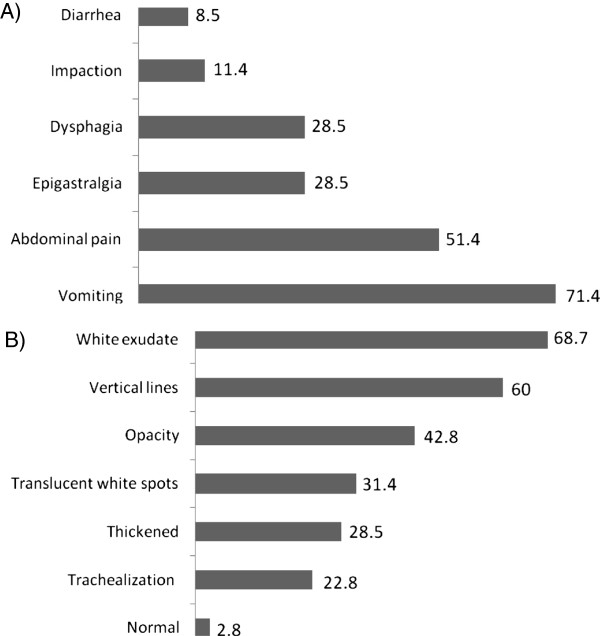
**Prevalence of symptoms and endoscopic findings in patients with EoE treated in HC-UFU. A.** Prevalence of symptoms findings in patients with EoE treated in HC **B.** Prevalence of endoscopic findings in patients with EoE treated in HC.

The most frequent symptoms were vomiting in 25 (71.4%) patients and abdominal pain in 18 (51.4%) patients. The endoscopies examinations revealed alterations in 34 (97.2%) patients, and the following alterations were the most common: loss of the vascular pattern with an adherent, white exudate (in 24 (68.5%) patients), vertical lines, and opalescent mucosa. The histological evaluation showed that the presence of EOS varied from 20 to 150 EOS/HPF, with a median of 40 EOS/HPF. The symptom data and the endoscopic findings are shown in Figure 1.

The 35 patients underwent an evaluation of the esophageal pH for 24 hours (pH monitoring) and values within normal ranges were observed in 26 (74.2%) patients.

The tests demonstrated a positive result for sensitivity to foods and/or aeroallergens in 27 (77.1%) of the evaluated patients. Among the sensitive patients, 24 (68.5%) were sensitive to at least one inhalant, and mites and dog epithelia were the most common sensitivities observed.

The skin prick test and the food atopy patch test data demonstrated 16 (45.7%) patients with sensitivity to foods. The foods with the highest frequency of sensitivity were cow’s milk, soybean, and chicken. The distribution of positivity in the tests is shown in Table 2.

**Table 2:** Distribution of the positivity of the tests performed to evaluate food sensitivity in patients with EoE

**Foods**	**APT**	**SPT**	**APT + SPT**
	**n(%)**	**n(%)**	**n(%)**
Cow’s Milk	4(11.4)	4(11.4)	8(22.8)
Soybean	6(17.1)	2(5.7)	8(22.8)
Chicken	4(11.4)	1(2.8)	5(14.2)
Fish	4(11.4)	1(2.8)	5(14.2)
Beef	4(11.4)	0(0.0)	4(11.4)
Pork	2(5.7)	1(2.8)	3(8.5)
Corn	2(5.7)	0(0.0)	2(5.7)
Egg White	1(2.8)	2(5.7)	3(8.5)
Wheat	1(2.8)	0(0.0)	1(2.8)

*SPT* skin prick test.

*APT* atopy patch test.

A negative result for every sensitivity test was observed in 8 (22.9%) patients. Significant differences related to the age ranges were not observed between the sensitive patients and the non-sensitive patients; however, statistically significant (p < 0.05) differences in the distribution of sex, clinical presentation, 24-hour pH monitoring, and the endoscopic results were observed between the 2 groups (Table 3).

**Table 3:** Comparative analysis of the descriptive data of the patients with EoE cared for at the HC-UFU who were both sensitized and non sensitized to the allergic tests performed

**Data**	**Non sensitized**	**Sensitized**	p-value*
	**n(%)**	**n(%)**	
Male sex	2(25.0%)	18(66.6%)	0.0194
Food impactation	0(0.0%)	4(14.8%)	0.0002
Vomiting	8(100%)	18(66.6%)	0.0002
Normal pH monitoring	8(100%)	17(62.9%)	0.0001
White exsudate	2(25.0%)	21(77.7%)	0.0023
Opacity	1(12.5%)	12(44.4%)	0.0347

*Mann Whitney test (significante p value <0.05).

## Discussion

EoE is a chronic inflammatory disease of the esophagus that has sparked the interest of doctors and scientists all around the world because of the increased number of cases in children and adults [1,17,18]. In this study, we investigated pediatric patients with EoE in our environment and determined whether our population exhibits the clinical characteristics and allergen sensitivities similar to the data reported in the literature.

Consistent with the majority of published studies involving patients with EoE, we observed a predominance of the disease in males [1,12,18,19]. The patients exhibited elevated prevalence of asthma, rhinitis, atopic dermatitis, and a high parental history of allergies, which is similar to observations reported in previous studies [1,2,18].

The reason for the lengthy time interval between the onset of the symptoms and the diagnosis of the disease observed in this study may be that EoE exhibits a symptomatology similar to other common diseases, such as GERD. This possibility has been reported in other studies in which the average time between the onset of the EoE symptoms and the first diagnostic endoscopy was 36 months [20]. However, other factors may be associated with the diagnostic delay, such as the doctors’ lack of knowledge of the disease and the need for specialized examinations, such as pediatric endoscopy and esophageal biopsies, which are performed only in referral centers.

We observed that only 2.8% of the endoscopies exhibited a normal appearance, which is in contrast to the results obtained in a retrospective study involving 381 children in which 32% of the endoscopic examinations of the patients with EoE were within the normal range [18]. The standardization of the endoscopic examination, combined with the routine performance of more than 1 biopsy and the fact that all of the examinations were performed by the same 2 professionals at the referral center, may have contributed to the higher diagnostic rates. Caution when routinely performing esophageal, stomach, and duodenum biopsies in all pediatric patients undergoing endoscopy has already been recommended in other studies [21,22].

The majority of the patients (82.8%) did not exhibit alterations in their growth curves at diagnosis, which is consistent with a study in which the patients with EoE were monitored for more than 10 years. In the study, a relevant nutritional impact was not observed in the majority of the individuals, even among those patients still exhibiting symptoms and not demonstrating histological remission [23]. However, the disease only affects the esophagus and spares other segments of the gastrointestinal tract, which could explain why the disease did not affect the growth curve of patients.

In our study, the majority of the patients were school-aged children and pre-adolescents with a median age similar to that of other studies [2,18,19]. In 1 of our patients, the diagnosis was performed at less than 24 months of age, and the refusal to eat made it necessary to perform a gastrostomy to maintain the nutritional status of the patient.

In the majority of our patients, the symptoms mimicked GERD, such as vomiting and abdominal pain, which are generally persistent even with long-term treatment with PPI, and this finding is currently considered a criterion for a clear diagnosis of EoE [1,18,24]. Similar to other reports, a large number of the patients who underwent the 24-hour pH monitoring evaluation exhibited and reflux index within the normal range, excluding the acidic exposure of the esophagus as a cause of EoE [11]. Currently, the concomitance of EoE and GERD is well-documented, which explains a number of the altered pH monitoring examinations; however, the patients do not exhibit a clinical or histological response to the PPI [1]. Dysphagia and sudden impaction in asymptomatic individuals have been reported in a significant number of patients, usually in older children and adults [25].

The physiopathology of EoE has not been completely described, and further information is needed to understand this disease. Currently, evidence supports the presence of an altered immune response as a possible causal factor of the disease; however, the existence of an associated antigen has not been confirmed [1]. The remission of the clinical and histological profile of EoE with the use of elemental diets is strong evidence for associating EoE with an food allergy [1,2,23,26]. In addition, other studies have demonstrated clinical and histological improvements using empirical dietary exclusions or dietary exclusions guided by allergy testing, suggesting that a food antigen may be responsible for the immune alteration described for EoE [12,16,25,27].

Despite reports in the literature of remission following different elimination diets, in the daily practice of primary care in public hospitals or private offices, a strict diet is difficult to apply and may have a major impact on the quality of life of the pediatric patients and their families [28]. Therefore, the use of allergy tests to evaluate the immediate and delayed allergic sensitivity to foods has been suggested by a number of authors, which allows the provision of a directed elimination diet with fewer repercussions in the daily and social life of these patients [2,5]. In our patients, the tests revealed fewer sensitivities to foods compared to other studies using the same techniques [12,16,29].

Because this report describes a cross-sectional study, it does not allow for the claim that sensitivity actually translates into food allergy. To prove this claim, it is necessary to perform a series of endoscopies and biopsies to demonstrate the role of each food in the genesis of the esophageal inflammatory process, which is recognized as the gold standard for establishing the relationship between EoE and food allergy [12].

Pertaining to the involvement of inhalants in the pathogenesis of EoE, an experimental study in rats using a placebo control demonstrated that esophageal hypersensitivity was simultaneous with the development of pulmonary inflammation, particularly in genetically modified animals that were eotaxin-3 and interleukin 5-positive, demonstrating that similar to asthma, sensitivity to inhalants might be involved in the pathogenesis of the disease [30]. Other studies suggest the participation of inhalants in the activation of the disease during seasons associated with high levels of pollen [31].

In our patients, there was a notable positivity for the sensitivity to inhalants, similar to what was observed in the prick test. We stress the need for further studies to understand the actual association of inhalants with EoE. Notably, a large proportion of the patients were carriers of other atopic diseases, such as asthma and rhinitis, and it was not possible to establish whether the positivity in the test was because of one of these diseases, EoE, or all of the above.

The observation that one group of patients exhibited allergen sensitivity and another group did not exhibit allergen sensitivity suggests that EoE, similar to asthma, exhibits different inflammatory patterns that lead to a common clinical presentation; therefore, it is possible for the disease to exhibit different phenotypes.

Whereas the cause of the immune alteration responsible for esophageal inflammation in EoE is still under discussion, we need to offer our patients the most effective treatment possible with the smallest impact on the quality of life. The consensus recommended treatment, in addition to an elimination diet, is the administration of topical corticosteroids that are swallowed, which was employed in all of the patients who participated in this study [1,2,32]. To realize the full impact of EoE in children, some authors note that the histological findings and professional perception are important for patient assessment through the development of quality of life questionnaires [33].

## Conclusions

In conclusion, the patients evaluated in this study exhibited clinical characteristics that were similar to other cases of EoE described in the literature, demonstrating an important association with other allergic diseases that also exhibit an alteration in the immune system’s expression. The need for future studies to gain further insight into the pathogenesis of this disease does not prevent us from offering a treatment that could ensure an improvement in the quality of life and reduce the chance of acute events related to EoE.

## Abbreviations

EoE: Eosinophilic esophagitis; EOS: Eosinophils; HPF: High power field; GERD: Gastroesophageal reflux disease; PPI: Proton-pump inhibitor; HC-UFU: Clinical Hospital of Federal University of Uberlandia.

## Competing interests

The authors declare that they have no competing interests.

## Authors’ contributions

ERMAR conceived of the study, and participated in patients’ evaluation and data analysis. CPB participated in patients’ evaluation and endoscopy analysis. LHY carried out allergic evaluation. ATS participated in nutritional patient’s evaluation and food preparation for patch tests. GRSS conceived of the study, and participated in its design and coordination. RMCP as data statistics analyst. All authors read and approved the final manuscript.
